# From Initial Nucleation to Cassie-Baxter State of Condensed Droplets on Nanotextured Superhydrophobic Surfaces

**DOI:** 10.1038/srep42752

**Published:** 2017-02-16

**Authors:** Cunjing Lv, Xiwen Zhang, Fenglei Niu, Feng He, Pengfei Hao

**Affiliations:** 1 Department of Engineering Mechanics, Tsinghua University, Beijing 100084, China; 2 Institute for Nano- and Microfluidics, Center of Smart Interfaces, Technische Universität Darmstadt, Darmstadt 64287, Germany; 3 State Key Laboratory of Alternate Electrical Power System with Renewable Energy Sources, North China Electric Power University, Beijing 102206, China

## Abstract

Understanding how droplet condensation happens plays an essential role for our fundamental insights of wetting behaviors in nature and numerous applications. Since there is a lack of study of the initial formation and growing processes of condensed droplets down to nano-/submicroscale, relevant underlying mechanisms remain to be explored. We report an *in situ* observation of vapor condensation on nano-/microtextured superhydrophobic surfaces using optical microscopy. An interesting picture of the vapor condensation, from the initial appearance of individual small droplets (≤1 μm) to a Cassie-Baxter wetting state (>30 μm), are exhibited. It is found that individual droplets preferentially nucleate at the top and the edge of single micropillars with very high apparent contact angles on the nanotextures. Scenarios of two distinguished growing modes are reported statistically and the underlying mechanisms are discussed in the view of thermodynamics. We particularly reveal that the formation of the Cassie-Baxter wetting state is a result of a continuous coalescence of individual small droplets, in which the nanotexture-enhanced superhydrophobicity plays a crucial role. We envision that these fundamental findings can deepen our understanding of the nucleation and development of condensed droplets in nanoscale, so as to optimize design strategies of superhydrophobic materials for a broad range of water-harvesting and heat-transfer systems.

There has been significant interest in developing superhydrophobic surfaces for promoting dropwise condensation. Such surfaces benefit greatly from the combination of nano-/microstructures and inherent hydrophobicity of their chemistry, which is able to attain extreme non-wetting properties with vapor trapped underneath to render coalescence-induced self-propelled dropwise condensation^1^,^2^,^3^,^4^,^5^,^6^,^7^,^8^,^9^,^10^,^11^,^12^,^13^,^14^. Understanding the development of vapor condensation and morphology of small droplets on superhydrophobic surfaces plays a significant role in a range of applications, such as thermal power industry^15^,^16^,^17^,^18^, air conditioning^19^, anti-fogging and anti-icing^20^,^21^, desalination^22^, and water-harvesting systems^23^,^24^.

This field of research has been very active for about twenty years, with theoretical, experimental and computational viewpoints. To date, various artificial superhydrophobic materials have been deliberately developed to sustain dropwise condensation over time to avoid a degradation of unwanted filmwise condensation (i.e., sticky Wenzel state)^4^,^11^,^14^,^25^,^26^,^27^. Meanwhile, a considerable amount of work has focused on dynamic condensation processes, such as wetting state transitions^8^,^9^,^10^,^12^,^14^,^15^,^28^, coalescence-induced jumping and sweeping^10^,^11^, spontaneous droplet removal and transport^1^,^2^,^29^,^30^, robustness of the superhydrophobicity during condensation^31^, etc. Since generally the heat transfer enhancement could greatly benefit from promoting dropwise condensation, in order to achieve this aim, more recently, researchers also dedicate attention from new perspectives rather than solely using superhydrophobic materials. For instance, the development of methods to enhance dropwise condensation of lower surface tension liquids for special industrial applications^32^, the strategy of using hybrid surfaces to improve multiple aspects of heat transfer properties (e.g., the droplet nucleation density, growth rate and self-removal)^4^, and the idea of designing surfaces covered with slippery asymmetric bumps to achieve unprecedented droplet growth and transport^1^. These approaches broaden the application regime of dropwise condensation to realize exceptional multifunctionalities. Previous researches of droplet condensation on textured surfaces mainly revealed the existence of three distinguished wetting states, i.e. Cassie-Baxter (CB), Wenzel and partially wetted (PW) states^25^,^28^,^33^. CB droplets are formed and suspend on top of the textures with the base area in a composite wetting state; the base of a Wenzel droplet is completely wetted; while a PW droplet is a composite of the CB and Wenzel states. It has to be noted that there is actually one more option, namely the Cassie-Baxter (CB) impregnating wetting regime^34^,^35^ (pores are filled with liquid, but solid “islands” ahead the droplet are dry) occurring in various experimental situations^34^,^35^,^36^, which we think is worth being further explored for condensation. The above wetting states are highly dependent on the physiochemical properties of the condenser surfaces. Moreover, the condensation process is essentially related to the three stages of a droplet “life” on a surface: nucleation, growth and departure. However, most of the microscopic measurements have been performed by using environmental scanning electron microscopy (ESEM) in low-pressure and low-vacuum environments, which are essentially different from the atmospheric conditions due to the unfavorable effect such as evaporation, wettability modification and liquid charging^37^,^38^,^39^,^40^. Usually, large field of view and small potential were chosen to minimize these undesirable effects, but it limits the ability of observation, makes the morphology of small droplets (≤5 μm) in the initial condensation stage difficult to be visualized in detail. To the best of our knowledge, a systematic investigation of the initial condensation process of small droplets (i.e., ≤1 μm) on textures has yet to be done, and a fundamental understanding of their growth and development in natural environment remains extremely limited. In this context, it is worth bridging the wetting phenomena from the initial appearance of droplets in nano-/submicroscale and microscale to a formation of a Cassie-Baxter state in macroscale, to shed new light on dropwise condensation.

In this work, by employing optical microscopy, we developed a simple method to observe *in situ* vapor condensation on micropillared surfaces with nanotextures under atmospheric environment. An interesting picture of condensation processes, from the initial appearance of individual droplets (≤1 μm), their growth and coalescence dynamics, to a Cassie-Baxter wetting state (>30 μm), are first reported. It is observed that in the initial stage of condensation, droplets preferentially attach to the top and the edge of individual micropillars, growing up with distinguished scenarios. We further report that the coalescence can be rendered by a different number of droplets, this behavior will not only lead to out-of-plane jumping motion, but also lead to a formation of suspended CB wetting states. A statistical analysis is performed and a quantitative model is developed which are expected to enhance our understanding of the underlying mechanisms. This study will contribute to design optimal structures/materials with robust superhydrophobicity and a realization of a broad range of practical applications such as water collection and heat dissipation.

## Experiments

**Figure 1:**
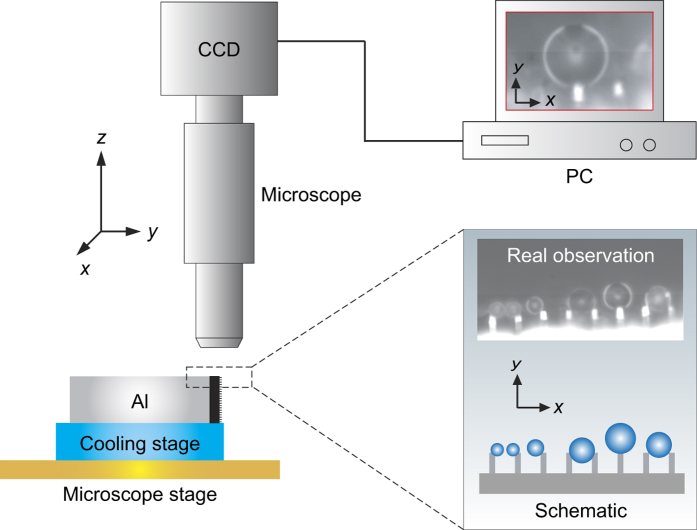
Schematics depicting the experimental setup. The silicon wafer with nanotextures on micropillars is vertically attached on one side of an aluminum block, which is fixed on a cooling stage under a microscope stage. The condensation processes are visualized from the top view (i.e., (−*z*)-direction) by using an optical microscope and recorded by a CCD camera. Insets in the schematics are selected frames (in the *xy*-plane) of the real experimental processes.

As shown in Fig. 1, the key part of our experimental setup is a combination of a CCD camera (ES2001, Radlake, USA) and an optical microscope (BX51, Olympus, Japan), which accounts for an *in situ* observations of the condensation under a moist ambient environment. The feature of this experiment is that the silicon wafer with nano-/microstructures was cut along the orientation of the micropillars into a narrow strip (1 mm × 20 mm), and then this strip was attached on one side of an aluminum block (20 mm × 20 mm × 2 mm), meanwhile, the side walls of individual micropillars with nanostructures were exposed directly to the objective lens (i.e., (−*z*)-direction in Fig. 1). We approximate the condensation observed from this side view as what occurs in the micropillared forest. Because of the small size of the droplets, the effect of gravity is negligible. The aluminum block is placed horizontally on a Peltier cooling stage, which is installed on the microscope stage with a resolution of 0.2 μm in the vertical motion. This resolution is sufficient high to enable the objective lens to focus on the interested region of the nano-/microstructures. The laboratory temperature is measured at 29 °C with a relative humidity of 40%. During running of the cooling system, the temperature of the specimen is well maintained at 10 ± 1 °C, and the condensation process captured by the CCD camera has a recording speed of 2.5 frames per second.

**Figure 2:**
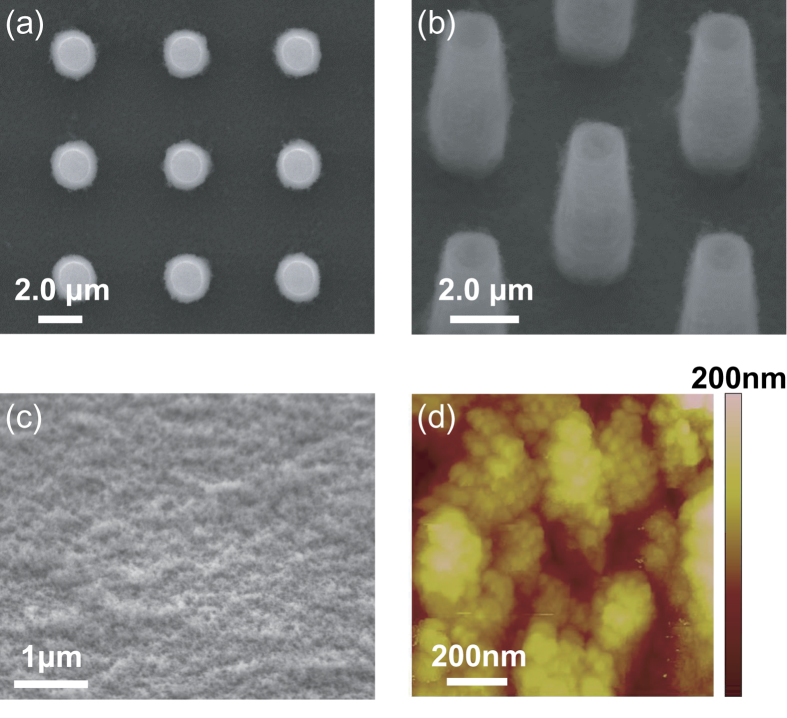
Topology of the Glaco-coated micropillars observed from the top view (**a**) and oblique side view (**b**) by employing SEM. The width, spacing and height of the micropillars are *L* = 1.8 μm, *S* = 4.8 μm and *H* = 5 μm, respectively. The zoomed regions displaying in (**c**) and (**d**) are characterized by SEM and AFM, respectively, showing a thin layer of superhydrophobic nanotextures consisting of hydrophobic colloids. The roughness is *Ra* = 24.5 nm, corresponding to a 1 μm × 1 μm area on a flat Glaco-coated silicon wafer.

The square-shaped micropillars are fabricated on the silicon wafer substrate with side length *L* = 1.8 μm, spacing *S* = 4.8 μm of the neighbors, and height *H* = 5 μm (Fig. 2a,b). These samples are treated with a commercial coating agent (Glaco Mirror Coat “Zero”, Soft 99, Co.)^41^,^42^, in which hydrophobic nanoparticles (Fig. 2c,d) are contained to guarantee excellent superhydrophobicity. Even on flat one-tier nanotextured surfaces (Fig. 2c), the apparent contact angle reaches as high as *θ* = 159.2° ± 1.5° (with a corresponding contact angle hysteresis Δ*θ* = 10.2° ± 2.1°). Figure 2c,d are performed on a flat Glaco-coated silicon wafer, demonstrating that the coating is composed of self-assembled nanoparticles with a fractal-type structure, whose space and depth range from about 100 nm to 300 nm and 50 nm to 200 nm, respectively. The roughness of this coating is *Ra* = 24.5 nm, which is characterized by employing AFM on a 1 μm × 1 μm area in nanometer level.

## Results

### Initial Condensation – Droplet Growing

**Figure 3:**
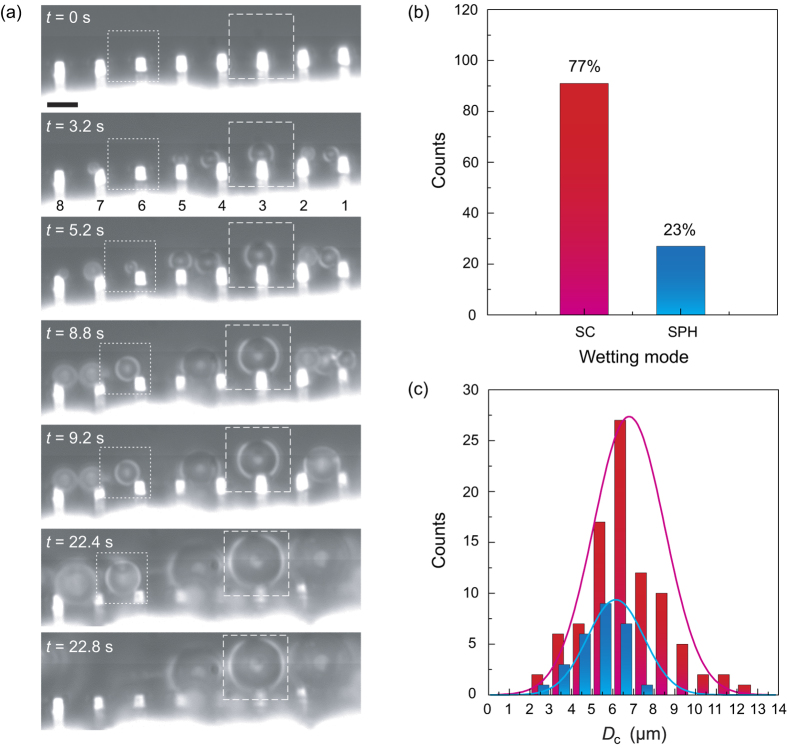
Initial growing processes of the droplets on the nano-/microstructured substrate. (**a**) On top of pillar No. 3, the droplet exhibits a SC wetting mode, while at the intersection of the top and the side walls of pillar No. 6, the droplet exhibits a SPH wetting mode. The scale bar represents 5 μm. The probability of the wetting mode (**b**) and distribution of the maximum droplet diameter (**c**) of the SC and SPH wetting modes based on the statistics of 118 condensed droplets. The solid lines in (**c**) are used to guide the eye.

Rather than the concepts of the CB and PW wetting states from a macro view^14^,^43^,^44^,^45^,^46^,^47^,^48^ – the diameter of the droplets (typically >10 μm) are much larger than the size of individual textures, here we focus on the initial appearance and growing behaviors of very small droplets. The size of the droplets (<5 μm) is comparable with the size of the micropillars, which has rarely been reported before. As displayed in Figs 1 and 3 (see Supplementary Movie 1), after our cooling system runs, plenty of small droplets start to form around the top of the micropillars. For a convenient description, the micropillars are numbered (from the right- to the left-hand sides) and the growing processes are highlighted in the closed boxes in Fig. 3a.

It is interesting that two different growing modes at the beginning of the vapor condensation are observed: (1) When nucleation accidently happens on the top of individual micropillars (i.e., pillar No. 3 in Fig. 3a, as marked by the square with dashed lines), the droplet always attaches to the top during the initial growing process. Even though the diameter of the droplet highly increases (i.e., 9 μm at 22.8 s, we define *t*_0_ = 0 s as the beginning of the condensation), the solid-liquid contact area does not change appreciably. Since the droplet is standing on the top of a single micropillar, we call this condensation behavior “individual spherical cap (SC) wetting mode”. (2) Meanwhile, we observe that some small droplets appear at the intersection of the top and the side walls of a single micropillar (i.e., pillar No. 6 in Fig. 3a, as marked by the square with dotted lines). Considering the solid-liquid contact regions of such droplets are very small and the droplets exhibit a spherical shape, we call it “individual spherical (SPH) wetting mode”. Even though the droplet exhibits an extremely water-repellent performance in these two wetting modes (i.e., a high apparent contact angle) resulting from the superhydrophobic nanotextures, it is hard to discern the wetting state on the solid-liquid contact area at the nanolevel. Strictly speaking, the contact area is in a state that either air can be trapped or not in the nanotextures^25^,^28^,^33^,^34^,^35^. It should be stressed that these phenomena are the unique features of this study, which is distinct from the previous observations of the constant contact line (CCL) or constant contact angle (CCA) mode for macrodroplets^49^. We statistically analyzed the condensation of 118 droplets, and give the probability of the wetting mode and the distribution of the maximum droplet diameter *D*_c_ in Fig. 3b and c, respectively. *D*_c_ is defined as the maximum size the droplet can reach before it coalesces with the neighboring droplets or touches the neighboring miropillars. It is found that the probability of the SC mode is more than three times higher than the SPH mode. The maximum diameter of the droplet ranges from 2 to 12 μm, with an average value of 6.6 ± 2.0 μm and 5.2 ± 1.2 μm for the SC (more counts) and SPH (less counts) modes, respectively.

**Figure 4:**
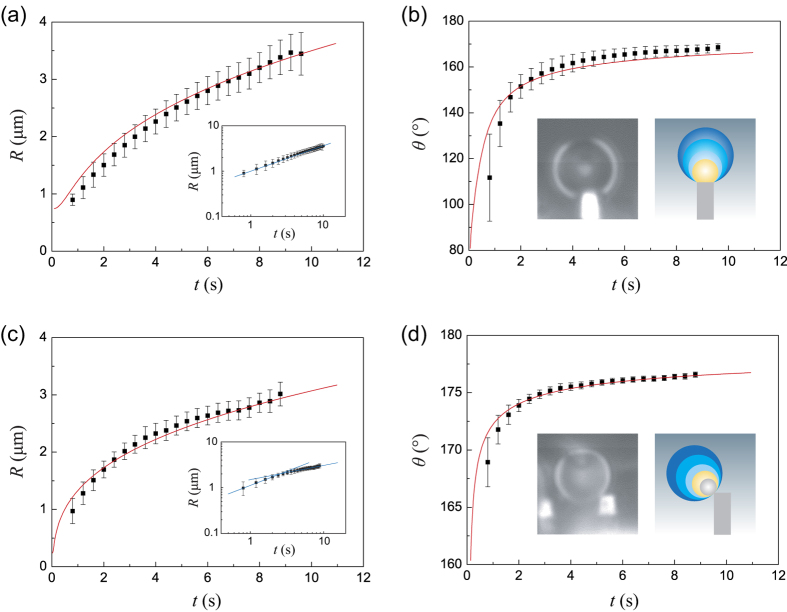
Instantaneous radii and apparent contact angles of the droplets as functions of time. (**a**,**b**) give the initial growing process of the SC mode, in which the instantaneous radius obeys *R* ~ *t*^0.56^. (**c**,**d**) show the initial growing process of the SPH mode, in which the instantaneous radius grows very fast (*R* ~ *t*^0.62^) in the first several second, while the growth velocity decreases to about half of the initial stage (*R* ~ *t*^0.31^). Each dot is the average value of ten measurements with the standard error (see Fig. S1). Insets in (**b**,**d**) include both the real experimental frames and schematics. The red solid lines in the normal plots are theoretical results using Eq. (4), whereas the blue lines in the log-log plots are direct fittings using the scaling law *R* ~ *t*^α^.

To gain further insights and quantify the growing processes, relationships of *R vs t* and *θ vs t* are given in Fig. 4, denoting *t, R* and *θ* the time, the instantaneous radius and the apparent contact angle of the droplet. Since the diameter *d* of the solid-liquid contact area and the height *h* of the droplet can be measured from experiments directly, *R* and *θ* are calculated using *R* = *d*^2^/8 *h* + *h*/2 and cos*θ* = 1 − 8*h*^2^/(*d*^2^ + 4*h*^2^), based on that the droplet is a spherical cap^50^. The results shown in Fig. 4 were performed using a statistical way, and each of them is the average value of ten measurements with the standard error (more details are shown in Fig. S1). In the SC mode (Fig. 4a and Supplementary Movie 1), when *d* ≤ *L* = 1.8 μm (typically when *t* ≤ 0.8 s), *d* is adopted using the real contact area; but when later *d* reaches the size of the micropillar, *d* = *L* = 1.8 μm is adopted to calculate the radius *R* of the droplet. During this process, the apparent contact angle *θ* we get ranges from 81° to 171° (see Fig. S1). In the SPH mode (Fig. 4c and Supplementary Movie 1), *d* = 0.36 μm is adopted. However, in the SPH, the apparent contact angle are very high, ringing from 167° to 177°. Moreover, it is found that the growing of the droplets obeys a scaling law *R(t*) ~ *t*^*α* ^1,12,14,51,52,53, denoting *α* the scaling exponent. For droplets in the SC, *α* ≈ 0.56. By contrast, it is very surprising that when droplets are in the SPH, two scaling laws are observed, *α* ≈ 0.62 in the very beginning but then it dramatically decreases to 0.31 at the subsequent stage.

### Droplet Coalescence – CB Droplet Formation

As the condensation process goes further, both a large number of coalescence-induced jumping and suspended CB droplets appear (i.e., 2 *R* > 10 μm), which was studied very often recently^10^. Unfortunately, an unequivocal picture from the nucleation to the formation of CB droplets remains obscure. An unexpected droplet coalescence and formation of CB wetting state are elaborated as follows, which is another feature of this work.

**Figure 5:**
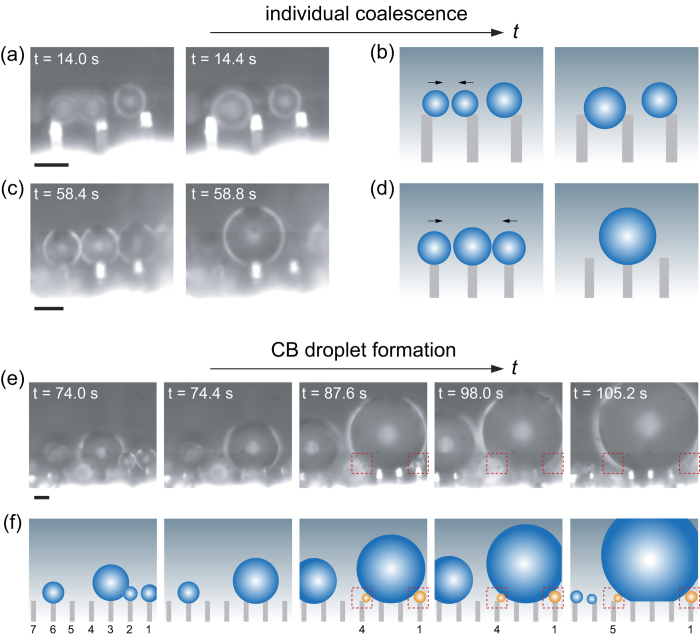
Coalescence of small individual droplets and the process of the formation of CB droplets. Two (**a**,**b**) or three (**c**,**d**) small droplets coalesce into bigger droplets suspended on top of the micropillars. (**e**) The formation of a CB droplet accomplished by continuously combination of individual droplets. The scale bar in each figure represents 5 μm, and the time scale shown in each figure corresponds to Supplementary Movie 1. Schematic plots are given in (**b**,**d**,**f**). The red dotted squares marked in (**e**) and (**f**) are employed to highlight the coalescence phenomena.

Figure 5a displays some interesting coalescence phenomena. Firstly, the droplets are too small, they just attach at the edges of the micropillars resulting from the solid-liquid area adhesion or the contact line pinning. When their size is big enough (2 *R* ≈ 4 μm), they touch each other and coalesce into one bigger droplet. Since they are still very small that the redundant surface energy released during coalescence cannot trigger jumping^12^, the bigger droplet suspends between the two neighboring micropillars. It should be stressed that such coalescence behavior can be also triggered by multiple droplets, e.g., by three small droplets when accidently they close to each other, as shown in Fig. 5c. In order to present the phenomena better, we give schematics in Fig. 5b,d. It is interesting to note that as the condensation goes on, the droplets always suspend on the top of the micropillars rather than go to the valley of the micropillars. This behavior would guarantee avoiding a risk of further development to the unwanted Wenzel wetting state when a considerable amount of liquid is accumulated, so as to sustain the robustness of the suerphydrophobicity which will account for the formation of CB droplets in the later stage. As the droplets grow bigger and bigger, numerous coalescence-induced jumping events easily happen, which suggests there is a high probability that the nanotextures of the solid-liquid contact area are trapped by air.

When a relative bigger droplet forms and suspends steadily on top of the micropillars, an interesting coalescence phenomenon arises: we clearly observe that other smaller droplets closely around continuously merge with this big one, with no time to grow up further. Schematic of the growing processes is given in Fig. 5f, the coalescence processes are highlighted using dotted red squares, which corresponds to the real case (i.e., Fig. 5e and Supplementary Movie 1). Specifically, these instantly disappeared droplets are small droplets on the top of pillar No. 1 and No. 4 during 74.8 s ~98 s, and pillar No. 1 and No. 5 during 98.8 s ~105.2 s (Note: we only depict the left seven micropillars in Fig. 5e,f compared with Movie 1). At some moment, the size of these disappeared droplets accounting for this coalescence behavior seems very small, that is, down to submicro scale. Driven by this behavior, the main droplet grows bigger and bigger, until a CB droplet is formed (~35 μm in diameter at 105.2 s). To the best of our knowledge, this is the first *in situ* exhibition of the growing and coalescence processes of individual small droplets to CB droplets under atmospheric conditions, and we envision our study will bridge the wetting phenomena in a large range of scales.

## Discussion

Underlying mechanisms of the above condensation behaviors can be understood in the light of thermodynamics. The heat transferred to the droplet form the vapor is assumed to be negligible as compared to the energy released *Q* = *H*_fg_*ρ*_L_*V(R, θ*) during the phase change process^50^,^54^, denoting *H*_fg_, *ρ*_L_ and *V(R, θ*) the latent heat of vaporization, mass density and instantaneous volume of the droplet, respectively. The heat transfer rate *q*_d_ through the droplet is equal to the rate at which the enthalpy of the newly condensed vapor changes^54^,


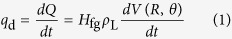


It is assumed that the base surface of the droplet is at a uniform temperature, so the total temperature drop between the vapor and the surface, i.e., Δ*T* = *T*_sat_ − *T*_surf_, is equal to the sum of temperature differences due to all the contributing thermal resistances^50^,^54^, denoting *T*_sat_ and *T*_surf_ the saturation temperature and vapor temperature, respectively. Namely, Δ*T* results from the following components: the thermal resistance of the vapor-liquid interface (Δ*T*_i_), the conduction across the droplet (Δ*T*_drop_), the capillary depression of the equilibrium saturation temperature (Δ*T*_cap_), and the conduction through the hydrophobic coating (Δ*T*_coat_) with thickness *δ*_coat_ and thermal conductivity *k*_coat_,


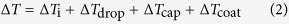


Related components are calculated using ∆*T*_i_ = *q*_d_/2*πh*_i_*R*^2^(1 − cos*θ*), ∆*T*_drop_ = *q*_d_*θ*/4*πRk*_L_ sin*θ*, ∆*T*_cap_ = 2*T*_sat_*σ*_LV_/*ρ*_L_*H*_fg_*R* = *R*_min_∆*T/R* and ∆*T*_coat_ = *q*_d_*δ*_coat_/*πR*^2^*k*_coat_ sin^2^*θ*, where *k*_L_ = 0.58 W m^−1^ K^−1^ is the water thermal conductivity (corresponding to *T* = 280 K), *σ*_LV_ = 0.073 N m^−1^ is the water-vapor surface tension, and *R*_min_ = 2*T*_sat_*σ*_LV_/*H*_fg_*ρ*_L_∆*T* is the critical radius of the condensed droplet. A substitution of these equations into Eq. (2) leads to,


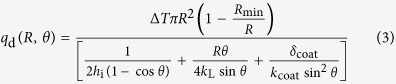


By combing Eqs (1) and (3), the droplet volumetric growth rate can be calculated as d*V(R,θ*)/d*t* = *q*_d_(*R,θ*)/*H*_fg_*ρ*_L_.

In the following, we will discuss two cases. In the case of a constant solid-liquid contact area (i.e., *d* is fixed during the condensation, as shown in Fig. 4a), we can get (d*V*/d*θ*) = π*d*^3^/8(1 + cos*θ*)^2^ through *V* = π(*d*^3^/sin^3^*θ*) (2 + cos*θ*)(1 − cosθ)^2^/24. Considering that d*V(R,θ*)/d*t* = (d*V*/d*θ*)(d*θ*/d*t*), a substitution of this equation into Eqs (1) and (3) leads to an ordinary differential equation:


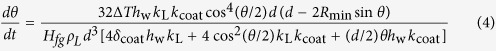


By contrast, in the case of a constant apparent contact angle *θ*, we can get (d*V*/d*R*) = π*R*^3^(1 − cos*θ*)^2^(2 + cos*θ*) through *V* = π*R*^3^ (2 + cos*θ*)(1 − cosθ)^2^/24. Considering that d*V(R, θ*)/d*t* = (d*V*/d*R*)(d*R*/d*t*), a substitution of this equations into Eqs (1) and (3) leads to


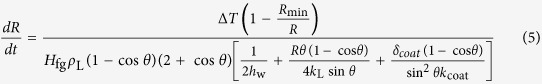


In our real experiments, the condensed droplets are supposed to be in the first case, so Eq. (4) is employed for comparisons with the experimental results. *T*_sat_ = 373 K, *H*_fg_ = 2257 KJ kg^−1^, *ρ*_L_ = 1000 kg m^−3^, *h*_w_ = 15.7 MW m^−2^ K^−1^ under 1.0 atm of pressure at 0 °C, *δ*_coat_ = 50 nm and *k*_coat_ = 16 W m^−1^ K^−1^, they are known and constant physical parameters. *d* = *L* = 1.8 μm is chosen for Fig. 4a,b. The only one unknown parameter is the temperature difference Δ*T*, which we could not measure exactly from the experiment. Here, Δ*T* = 0.03 K is adopted for a best fitting, and this value is quantitatively consistent with ref. 50. The comparisons (the red lines) in Fig. 4a,b suggest that this theory agrees with the experimental data. Furthermore, we also make quantitative analyses for the SPH, as shown in Fig. 4c,d. In this case, *d* = 0.36 μm is chosen based on real experiments (Fig. 4c and Supplementary Movie 1). However, in this case, Δ*T* = 0.1 K has to be adopted for a best fitting. Even though the results shown in Fig. 4 are based on a statistical way, the gap of Δ*T* between the two wetting modes (i.e. SC and SPH) indicates that there are some uncertainties exist and need to be further investigated. Very recently, some researches revealed that curvature, edges, corners and boundaries of the substrates have a strong effect on the vapor concentration profile and mass diffusion around the droplet^1^,^55^, these influences might be responsible for the inconsistency between the theory and experiments. How to experimentally distinguish the thermal resistance from each part of Δ*T* remains an open question, but is significant for us to figure out these uncertainties.

In order to gain more insight of the kinematic behaviors, we make further theoretical analyses about the scaling laws *R* ~ *t*^*α*^ of the growth of breath figures. Previous experimental and theoretical studies suggest that *α* typically ranges from 0 to 1 depending on the wettability of the substrate, wetting state of the droplet (e.g., individual droplet growth, coalescence), substrate dimensions, and working conditions of the condenser^14^ (Supplementary Table S1). Very recently, nucleation and coalescence on slippery surfaces show that *α* can not only be discontinuous, but it can also reach a surprisingly high value, i.e., *α* ~ 6.4^1^.

Compared with the conduction across the drop (Δ*T*_drop_), the contribution from the capillary depression of the equilibrium saturation temperature (Δ*T*_cap_), the thermal resistance of the vapor-liquid interface (Δ*T*_i_) and the conduction through the hydrophobic coating (Δ*T*_coat_) are much less (see Supplementary Information). For a simple analysis, we ignore Δ*T*_cap_, Δ*T*_i_ and Δ*T*_coat_. In the following, two possibilities are discussed. For the case of a constant solid-liquid contact area, considering *d* = 2*R* sin*θ*, d*R*/d*θ* = −*d* · cos*θ*/(2sin^2^*θ*) and d*R*/d*t* = (d*R*/d*θ*)·(d*θ*/d*t*), Eq. (4) can be written into,


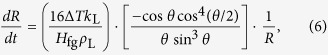


In our experiments, the droplets grow with a very high value of contact angles, we notice that when *θ* → 180°, [−cos*θ* · cos^4^(*θ*/2)]/(*θ* · sin^3^*θ*) → *d*/(32π*R*), which indicates d*R*/d*t* ~ 1/*R*^2^ and suggests *R* ~ *t*^1/3^. By contrast, for the case of a constant apparent contact angle, Eq. (5) can be written into,


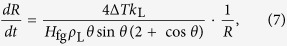


which indicates d*R*/d*t* ~ 1/*R* and *R* ~ *t*^1/2^. These exponents are generally consistent with previous theories^51^,^52^,^53^. Deviations between these scaling law analyses and the real experiments could be attributed that, on the one hand, the resolution of our microscopy technique is still not high enough to detect details (i.e., the contact angle and contact diameter) during the nucleation, so it is not easy to completely distinguish Eq. (6) from Eq. (7) in real experiments; on the other hand, contact angle hysteresis (i.e., line pinning), edge effect, the perturbation of the temperature and humidity around the micropillars could also affect the growing dynamics, as discussed very recently^1^,^55^. The above discussions will enrich our understanding of the underlying mechanism of condensation from different views. Carrying out further investigations both experimentally and theoretically to address these open questions is our next aim.

## Conclusions

In this paper, we studied the initial condensation behaviors of very small droplets in a saturation vapor pressure, as well as the growing process of the formation of CB droplets on nano-/microscaled two-tier structures using an *in situ* observation technology and a statistical way. We revealed that individual droplets preferentially nucleate at the top or the edge of the micropillars with a very high apparent contact angle and various growing rates. Furthermore, it is the first time we exhibit the whole landscape of condensation process, from the very initial appearance of individual droplets (≤1 μm) to a CB wetting state (>30 μm) as a result of continuously coalescence of individual small droplets. These nanotextures play an essential role, they not only enhance the robustness of superhydrophobicity under condensation, but also accelerate the rate of nucleation to form dropwise condensation and CB droplets. We speculate these findings to be crucial to deepen our understanding of condensation, and be useful for rapid cooling, water collection and anti-icing.

## Methods

### Fabrication of nanotexture-enhanced superhydrophobic surfaces

Silicon wafer substrates with square-shaped micropillars are fabricated by photolithography and etching of inductively coupled plasma (ICP)^6^,^7^. Then, they are produced by treatment of a commercial coating agent (Glaco Mirror Coat “Zero”, Soft 99, Co.) containing nanoparticles and organic reagent^41^,^42^. The superhydrophobic coating was applied on the substrates by pouring the Glaco liquid over them. A thin liquid film wets the substrates and dries in less than 1 min. The substrates are then put into an oven and kept at 200 °C for 30 min. The pouring and heating processes are typically performed three to four times.

### Surface characterization

Surface topographies are analyzed using a scanning electron microscopy (SEM, JSM 6330 from JEOL) and an atomic force microscope (AFM, NanoScope 5 from DI). Apparent contact angles and the contact angle hysteresis of the samples were measured and analyzed using a microgoniometer (JC2000CD1).

### Experimental setup

All of the experiments are performed using an optical microscopy technique under a moist ambient environment, allowing for focusing on the *in situ* dynamic character of the vapor condensation. Silicon wafer with nano-/microstructures is firstly carefully cut along the orientation of the micropillars into a narrow strip (1 mm × 20 mm), after that, the strip is attached on one side of an aluminum block (20 mm × 20 mm × 2 mm). Make sure that the side walls of individual micropillars with nanostructures are exposed to the objective lens. The aluminum block is placed horizontally on a Peltier cooling stage, which is installed on the microscope stage with a resolution of 0.2 μm in vertical motion. The laboratory temperature is measured at 29 °C with a relative humidity of 40%. During the running of the cooling system, the temperature of the sample is well maintained at 10 °C ± 1 °C. Top-down imaging of the process is captured using a CCD camera (ES2001, Radlake, USA, with a recording speed of 2.5 frames per second) installed on an optical microscope (BX51, Olympus, Japan).

## Additional Information

**How to cite this article**: Lv, C. *et al*. From Initial Nucleation to Cassie-Baxter State of Condensed Droplets on Nanotextured Superhydrophobic Surfaces. *Sci. Rep.* **7**, 42752; doi: 10.1038/srep42752 (2017).

**Publisher's note:** Springer Nature remains neutral with regard to jurisdictional claims in published maps and institutional affiliations.

## Supplementary Material

Supplementary Information

Supplementary Information
