# Supplementary material for: From Initial Nucleation to Cassie-Baxter State of Condensed Droplets on Nanotextured Superhydrophobic Surfaces
Source: Sci Rep. 2017 Feb 16;7:42752. doi: 10.1038/srep42752 (PMC5311920; doi:10.1038/srep42752)
Supplement: Supplementary Information [file srep42752-s2.pdf]

Supplementary Information

From Initial Nucleation to Cassie-Baxter State of Condensed Droplets on Nanotextured Superhydrophobic Surfaces

Cunjing Lv[1,2], Xiwen Zhang[1], Fenglei Niu[3], Feng He[1] & Pengfei Hao[1*]

[1]Department of Engineering Mechanics, Tsinghua University, Beijing 100084, China

[2]Institute for Nano- and Microfluidics, Center of Smart Interfaces, Technische Universität Darmstadt, Darmstadt 64287, Germany

[3]State Key Laboratory of Alternate Electrical Power System with Renewable Energy Sources, North China Electric Power University, Beijing 102206, China

S1. Droplet growth in the initial condensation

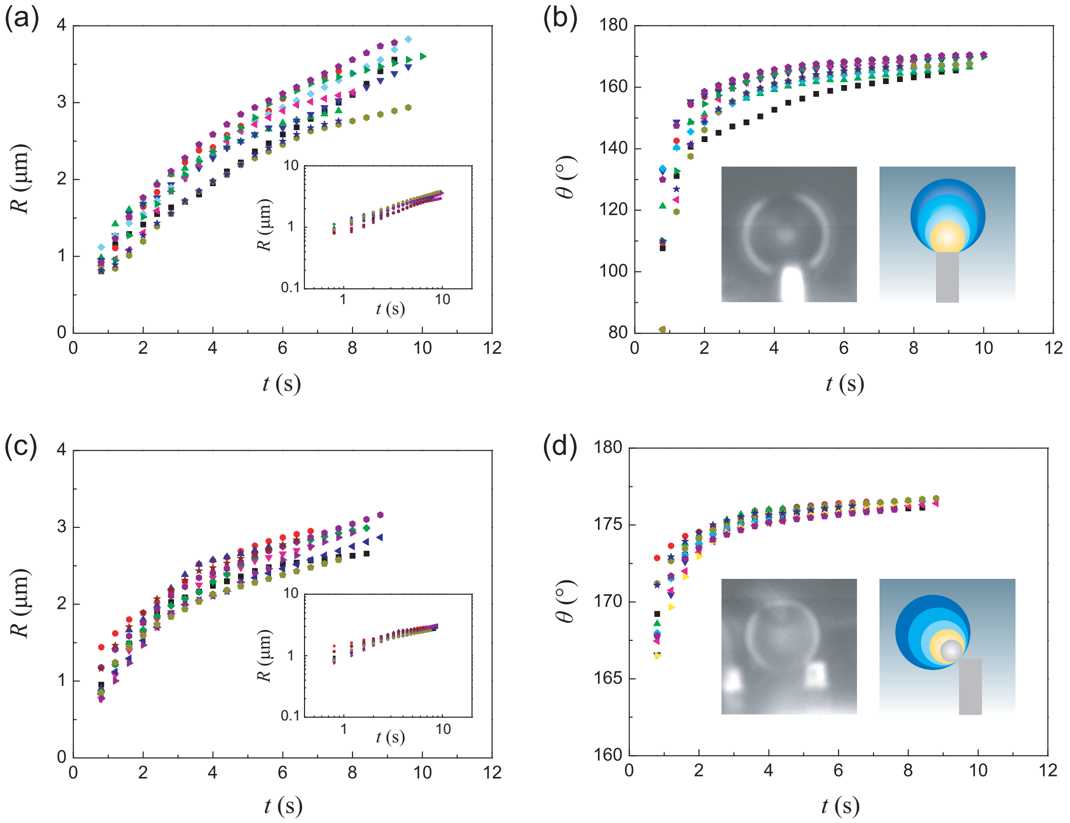

Figure S1. Statistical results of the wetting behaviors of the droplets in condensation. Dependence of the instantaneous radii and apparent contact angles of the droplets on the time for the SC (a)(b) and SPH (c)(d) wetting modes, respectively. Each test is based on ten experimental measurements. Relationships between *R* and *t* are displayed in the log-log plots in the insets of (a) and (c).

* To whom correspondence should be addressed. E-mail: haopf@tsinghua.edu.cn

S2. Contribution of the thermal resistances

On the basis of Eq. (4),

$$\frac{d\theta}{dt}=\frac{32\Delta T h_{\mathrm{w}} k_{\mathrm{L}} k_{\mathrm{coat}} \cos^4(\theta/2) d\left(d-2R_{\min}\sin\theta\right)}{H_{\mathrm{fg}}\rho_{\mathrm{L}} d^3\left[4\delta_{\mathrm{coat}} h_{\mathrm{w}} k_{\mathrm{L}}+4\cos^2(\theta/2) k_{\mathrm{L}} k_{\mathrm{coat}}+(d/2)\theta h_{\mathrm{w}} k_{\mathrm{coat}}\right]}, \tag{4}$$

we make comparisons to clarify the influences on the temperature difference (ΔT) resulting from each part of the contributing thermal resistance (i.e., ΔT_{i}, ΔT_{drop}, ΔT_{cap}, and ΔT_{coat}). The physical parameters are chosen as the same as shown in Fig. 4a,b (i.e., k_{L} = 0.58 W m^{-1} K^{-1}, T_{sat} = 373 K, H_{fg} = 2257 KJ kg^{-1}, ρ_{L} = 1000 kg m^{-3}, ΔT = 0.03 K, δ_{coat} = 50 nm, h_{w} = 15.7 MW m^{-2} K^{-1}, and k_{coat} = 16 W m^{-1} K^{-1}). In Fig. S2a,b, three cases are discussed: (1) the red solid line is the solution of Eq. (4) with all the contributing thermal resistances (i.e., $\Delta T = \Delta T_{\mathrm{i}} + \Delta T_{\mathrm{drop}} + \Delta T_{\mathrm{cap}} + \Delta T_{\mathrm{coat}}$). Furthermore, we give the contribution of each part of the thermal resistance to ΔT and q_{d} in Fig. S1c and Fig. S1d, respectively; (2) the blue dashed line ($\Delta T = \Delta T_{\mathrm{drop}} + \Delta T_{\mathrm{cap}}$) represents the thermal resistances are partially considered; (3) and the green dotted line ($\Delta T = \Delta T_{\mathrm{drop}}$) represents that we just consider the contribution from the conduction across the droplet.

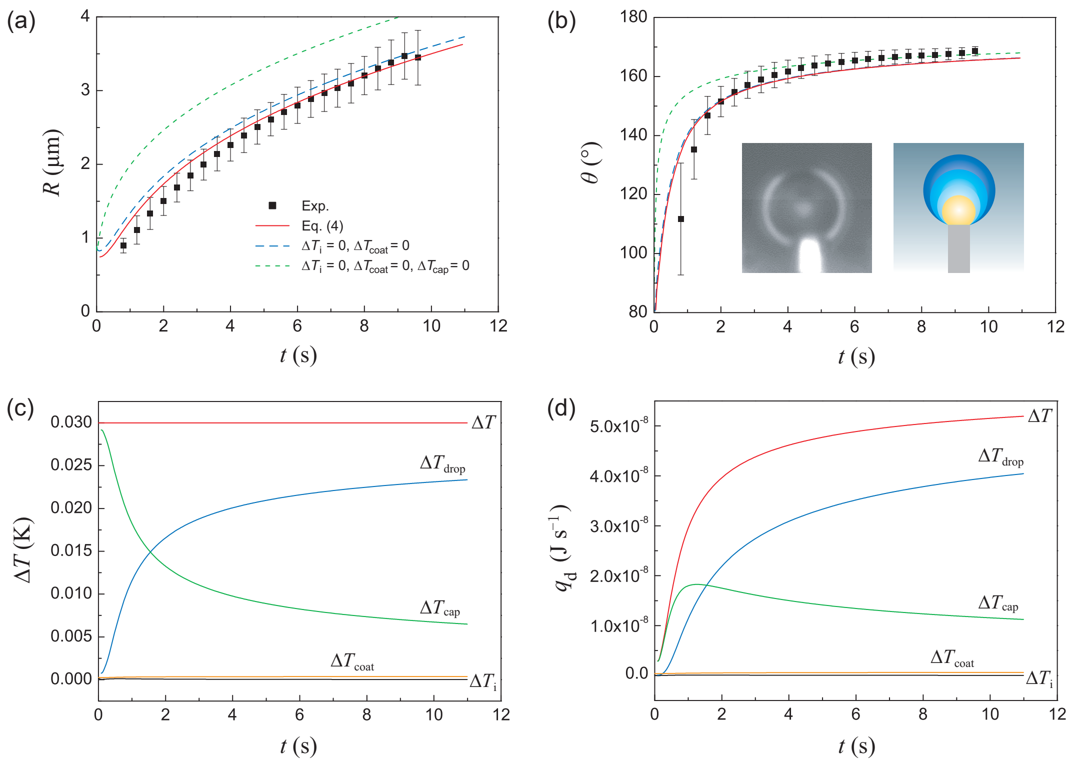

Figure S2. Instantaneous radii, apparent contact angles, temperature different and heat transfer rate of the droplets as functions of time. In (a)(b), different parts of the thermal resistances are given, the red

solid, blue dashed and green dotted lines represent Eq. (4) with $\Delta T = \Delta T_i + \Delta T_{drop} + \Delta T_{cap}$, $\Delta T = \Delta T_{drop} + \Delta T_{cap}$ and $\Delta T = \Delta T_{drop}$, respectivley. (c)(d) show the contribution of each part of the thermal resistance (when $\Delta T = \Delta T_i + \Delta T_{drop} + \Delta T_{cap}$) to the temperature difference ΔT and heat transfer rate q_d, respectively.

Fig. S2c,d suggest that the contribution from the thermal resistance of the vapor-liquid interface (ΔT_i) and the conduction through the hydrophobic coating (ΔT_{coat}) are very small. If we ignore ΔT_i and ΔT_{coat}, we can get,

$$\frac{dR}{dt} = \left(\frac{16\Delta T k_L}{H_{fg}\rho_L}\right)\cdot\left[\frac{-\cos\theta\cos^4(\theta/2)}{\theta\sin^3\theta}\right]\cdot\frac{1}{R}\cdot\left(1-\frac{R_{min}}{R}\right). \quad \text{(S1)}$$

S2. Scaling law analysis

When the constant contact area is fixed, considering during growth of a droplet, $R \gg R_{min}$ (which indicates that ΔT_{coat} is dominant in ΔT, as shown in Fig. S2c,d), so Eq. (S1) will further degenerate into,

$$\frac{dR}{dt} = \left(\frac{16\Delta T k_L}{H_{fg}\rho_L}\right)\cdot\left[\frac{-\cos\theta\cos^4(\theta/2)}{\theta\sin^3\theta}\right]\cdot\frac{1}{R}. \quad \text{(6)}$$

The relationship $d = 2R\sin\theta$ suggests,

$$\theta = \pi - \arcsin\left(\frac{d}{2R}\right). \quad \text{(S2)}$$

If the apparent contact angle is very high, i.e., when $\theta \to 180°$, on the basis of Eq. (S2), we get,

$$\left[\frac{-\cos\theta\cos^4(\theta/2)}{\theta\sin^3\theta}\right]\Bigg|_{\theta\to 180^\circ} \approx \frac{d}{32\pi}\cdot\frac{1}{R} + O\left(\frac{1}{R^2}\right), \quad \text{(S3)}$$

which indicates $dR/dt \sim 1/R^2$, and we can get $R \sim t^{1/3}$.

By contrast, when the apparent contact angle is fixed, if we assume that the contributions of ΔT_i, ΔT_{cap}, and ΔT_{coat} are very small compared with ΔT_{drop}, Eq. (5) will degenerate into,

$$\frac{dR}{dt} = \frac{4\Delta T k_L}{H_{fg}\rho_L\theta\sin\theta(2+\cos\theta)}\cdot\frac{1}{R}. \quad \text{(S4)}$$

Due to the constant value of θ, we get $dR/dt \sim 1/R$, which suggests $R \sim t^{1/2}$.

S3. Scaling exponents in previous experimental studies

Table S1. Selected scaling exponents of $R \sim t^{\alpha}$ from past studies.

Substrate	Wetting property	Situation	α	Refs.
Smooth glass	Hydrophobic	Immobile<br>Coalescence	**1/4**<br>**3/4**	1
Smooth glass<br>Iris leaves/grooved surfaces	Hydrophobic<br>Hydrophobic/super hydrophobic	Immobile<br>Interaction on the fibers<br>Interaction on the plane	**~ 1/3**<br>**~ 1/2**<br>**~ 1**	2,3
Smooth glass	Hydrophobic	Coalescence	**~ 1/4**	4
Smooth	Partially wetting	Individual droplet growth	**~ 1/4**	5
Smooth	Hydrophobic	Individual droplet growth	**1/2, 1**	6
Smooth<br>Two-tier surfaces	Hydrophobic<br>Superhydrophobic<br>Superhydrophobic	Immobile coalescence<br>Immobile coalescence<br>Mobile coalescence	**0.74 ± 0.05**<br>**0.82 ± 0.07**<br>**0.02 ± 0.04**	7
Nanostructured	Superhydrophobic	Partial wetting<br>Suspended<br>Coalescence	**0.78 ± 0.02**<br>**0.46 ± 0.03**<br>**0.05 ± 0.15**	8
Two-tier surfaces	Hydrophobic/super hydrophobic	Mobile coalescence	**0.5 ~ 0.6**<br>**~ 0.71 ± 0.05**	9
Papillae on lotus leaf	Superhydrophobic	Individual droplet growth	**~ 0.43**	10
Two-tier surfaces	Superhydrophobic	Individual droplet growth (constrained)	**~ 0.47**	11
Slippery surface	Hydrophilic bumps with hydrophobic surroundings	Mobile coalescence	**~ 0.82**<br>**~ 6.4**	12

S4. Movie 1

The video showing the development from the initial nucleation to the formation of Cassie-Baxter droplets, various wetting phenomena such as growth of small individual droplets, coalescence on top of the micropillars and coalescence-induced out-of-plane jumping are displayed.

References

1. Beysens, D. & Knobler, C. M. Growth of breath figures. *Phys. Rev. Lett.* **57**, 1433 (1986).
2. Beysens, D. Dew nucleation and growth. *C. R. Phys.* **7**, 1082–1100 (2006).
3. Narhe, R. D. & Beysens, D. A. Nucleation and growth on a superhydrophobic grooved surface. *Phys. Rev. Lett.* **93**, 076103 (2004).

4. Fritter, D., Knobler, C. M. & Beysens, D. Expeirments and simulation of the growth of droplets on a surface (Breath figures). *Phys. Rev. A* **43**, 2858-2869 (1991).
5. Rogers, T. M., Elder, K. R. & Desai, R. C. Droplet growth and coarsening during heterogeneous vapor condensation. *Phys. Rev. A* **38**, 5303 – 5309 (1988).
6. Leach, R. N., Stevens, F., Langford, S. C. & Dickinson, J. T. Dropwise condensation: experiments and simulations of nucleation and growth of water drops in a cooling system. *Langmuir* **22**, 8864-8872 (2006).
7. Boreyko, J. B. & Chen, C.-H. Self-propelled dropwise condensate on superhydrophobic surfaces. *Phys. Rev. Lett*. **103**, 184501 (2009).
8. Miljkovic, N., Enright, R. & Wang, E. N. Effect of droplet morphology on growth dynamics and heat transfer during condensation on superhydrophobic nanostructured surfaces. *ACS Nano* **6**, 1776-1785 (2012).
9. Jonathan B., Boreyko, C. & Collier, P. Delayed frost growth on jumping-drop superhydrophobic surfaces. *ACS Nano* **7**, 1618-1627 (2013).
10. Lv, C., *et al.* Condensation and jumping relay of droplets on lotus leaf. *Appl. Phys. Lett.* **103**, 021601 (2013).
11. Lv, C., Hao, P., Zhang, X. & He, F. Dewetting transitions of dropwise condensation on nanotexture-enhanced superhydrophobic surfaces, *ACS Nano* **9**, 12311-12319 (2015).
12. Park, K.-C., *et al.* Condensation on slippery asymmetric bumps. *Nature* **531**, 78-82 (2016).
